# PW01-028 – Developing a new severity score for FMF

**DOI:** 10.1186/1546-0096-11-S1-A81

**Published:** 2013-11-08

**Authors:** E Demirkaya, C Acikel, A Gul, M Gattorno, E Ben-Chetrit, H Ozdogan, P Hashkes, A Polat, O Karadag, A Livneh, S Ozen

**Affiliations:** 1 Pediatric Rheumatology, Gulhane Military Medical Academy, Ankara, Turkey; 2 FMF Arthritis Vasculitis and Orphan Disease Research Center, Gulhane Military Medical Faculty, Ankara, Turkey; 3 Rheumatology, Istanbul University, Istanbul, Turkey; 4 Pediatric Rheumatology, Ospedale Gaslini, Genoa, Italy; 5 Rheumatology, Hadassah-Hebrew University Medical Center, Jerusalem, Israel; 6 Pediatric Rheumatology, Shaare Zedek Medical Center, Jerusalem, Israel; 7 Rheumatology, Hacettepe University, School of Medicine, Ankara, Turkey; 8 Medicine, Sheba Medical Centre, Ramat-Gan, Israel; 9 Pediatric Rheumatology, Hacettepe University, School of Medicine, Ankara, Turkey

## Introduction

Severity is a basic feature that defines the prognosis of a disease. FMF presents with a variety of clinic and laboratory manifestations affecting severity, and evaluation of prognosis is an elusive task. Different severity scores have been previously proposed for FMF, and commonly used ones include Mor, Pras and Tel Hashomer severity scores however recent studies showed that there is no consistency among them.

## Objectives

The aim of this study was to develop and validate a new set of criteria for the assessment of disease severity for FMF patients by a multicenter study.

## Methods

Delphi survey technique was used for the initial phase of the study. A panel of experts including twenty-four FMF specialists from 16 countries participated in the survey. The first Delphi round aimed to identify all clinical and laboratory features considered to be associated with the severity of FMF. In the second round, 33 structured questions were developed to collect expert opinions about FMF severity. At the third and the last rounds, the expert panel was asked to evaluate the answers given to the questions in the previous round. After all rounds, a subgroup of the expert panel (ten experts and one facilitator) gathered in a consensus meeting (NGT) on November 13, 2012 in Washington DC, USA. In this meeting, the results of all previous rounds and items for the candidate criteria and their standard definitions were discussed.

## Results

In Delphi rounds, three of the mostly reported clinical items were ‘response to colchicine treatment’, ‘frequency of attacks’ and ‘presence of arthritis’ and laboratory parameters were high levels of Serum amyloid A (SAA), C-reactive protein (CRP), and the MEFV gene mutations. At the consensus meeting, items revealed through the second and the third rounds were reevaluated, and the following nine items were selected for the final set of severity criteria: presence of a chronic sequel (including amyloidosis, growth retardation, anemia, splenomegaly), organ dysfunction (nephrotic-range proteinuria), organ failure (cardiac, renal, thyroid etc.), frequency of attacks (average number of attacks between 1-2 per months), increased acute phase reactants (CRP, SAA, ESR, fibrinogen) during the attack-free period (at least 2 weeks after the last attack in 2 occasions one-month apart), involvement of more than 2 sites during an individual acute attack (pericarditis, pleuritis, peritonitis, synovitis), more than 2 different types of attack during the course of the disease (isolated fever, pericarditis, pleuritis, peritonitis, synovitis, erysipelas like erythema, scrotal involvement, vaginitis, myalgia etc.), duration of attacks (more than 72 hours in at least 3 attacks during one year), effort-induced leg pain (pain following standing or excercising, excluding other causes).

## Conclusion

The panel of experts agreed on the nine items to be used in the severity criteria for FMF, and results of the validation study are being waited for final definition and weights of the items.

## Disclosure of interest

None declared

